# The Origin of Unpleasant Aftertastes in Synthetic Sweeteners: A Hypothesis

**DOI:** 10.3389/fmolb.2018.00119

**Published:** 2019-01-18

**Authors:** Waldo Acevedo, Piero A. Temussi

**Affiliations:** ^1^Institute of Chemistry, Pontificia Universidad Católica de Valparaíso, Valparaíso, Chile; ^2^Dipartimento di Chimica, Universita' di Napoli Federico II, Napoli, Italy; ^3^Department of Basic and Clinical Neurosciences, King's College London, London, United Kingdom

**Keywords:** taste receptors, sweeteners, umami, bitter, docking

## Abstract

Most sweeteners are plagued with unwanted unpleasant aftertastes. Here we examined the possibility that one of the main reasons for this is the similarity of sweet and umami receptors. We performed docking calculations on models of sweet and umami receptors using as template the recently determined solid state structure of the first taste receptor, the medaka fish T1R2-T1R3 receptor. Our results show convincingly that sweeteners can be recognized also by the T1R1-T1R3 umami receptor, owing to the similarity of its architecture to that of the sweet receptor. We hypothesize that the T1R1-T1R3 receptor plays a key role in modulating the quality of sweet tastants, hinting at a simple explanation of their aftertaste. The prevailing ideas on taste coding favor strict labeling of taste cells, which would exclude that umami receptors can recognize other taste sensations. If some cross-talk based on the combinatorial model of taste is accepted, some sweet ligands can exert a bitter sensation. However, even if cross-talk is not admitted, direct stimulation of the umami receptor is bound to cause an aftertaste incompatible with good sweet quality.

## Introduction

The sweet taste of most sweeteners, notably the synthetic ones, is perceived as inferior to that of sucrose. The “quality” of sweet taste of many sweeteners is apparently jeopardized by the insurgence of other tastes after the main sweet sensation. The most common of these aftertastes is bitter. The explanation that immediately comes to mind is that these sweet compounds are recognized not only by the sweet receptor but also by other (taste) receptors.

Among human senses, taste is deceitfully simple because there are only five recognized taste sensations: sour, salty, sweet, bitter, and umami. Numerous ion channels present in the plasma membrane have been proposed as transducers for sour taste whereas it still unclear which cells transduce sodium chloride (Roper and Chaudhari, 2017). The last three tastes, related to food acceptance, are very well characterized together with the corresponding receptors (Chandrashekar et al., 2006). In Nature there is a very high number of sweet molecules, but they are all recognized in humans by a single receptor, a class C GPCR heterodimer composed of two similar peptide chains, called T1R2 and T1R3. On the contrary, a similar large number of bitter substances requires several class A GPCRs for recognition (Chandrashekar et al., 2006); these receptors are collectively called T2Rs. The third taste connected to food acceptance is umami, a relatively recent addition which characterizes recognition of L-amino acids and also sapidity (Chandrashekar et al., 2006; Temussi, 2009). This receptor, like that of sweet taste, is a class C GPCR heterodimer composed of two similar peptide chains, one (T1R3) identical to that of the sweet receptor and another (T1R1) similar to T1R2.

As suggested by Zhao et al. (2003), the taste quality of several sweeteners apparently stems from a combination of cells tuned to different taste modalities. In particular, considering the repellent nature of bitter taste, many researchers have tried to establish whether some sweeteners are also recognized by one or more bitter taste receptors (for instance, see Kuhn et al., 2004; Hellfritsch et al., 2012). In support of this hypothesis, Acevedo et al. (2016) have shown by *in silico* docking that Steviol glycosides can indeed interact strongly with two bitter receptors, specifically the hT2R4 and hT2R14 receptors.

In addition, it is worth recalling that long before any taste receptor was discovered it was common belief that sweet and bitter tastes had to be closely correlated (Verkade, 1968). This view originated mainly from the observation that many sweet tastants have an isomeric bitter counterpart. After the discovery of the actual sweet and bitter taste receptors, the idea that there had to be a similarity among sweet and bitter receptors was completely abandoned, because, as mentioned above, bitter molecules are recognized by several, similar class A (or F) GPCRs whereas all sweet molecules are recognized by a single receptor, i.e., class C GPCR heterodimer T1R2-T1R3. It became accepted instead that stochastically some sweetener might be recognized by one or more bitter receptors (Kuhn et al., 2004; Hellfritsch et al., 2012). This occurrence is not impossible. It certainly can explain some (after) tastes. However, it does not explain some of the subtlest pairs of very similar compounds in which one member (of the pair) is sweet and the other is bitter. These pairs include positional isomers, congeners, conformers and even enantiomers (Temussi, 2009). Certainly, it is difficult to accept that pairs of chiral isomers interact by chance with completely different receptors and give rise to similar quantitative responses: e.g., D-Trp is very sweet whereas L-Trp is very bitter.

A way out of this dilemma was suggested by Temussi (2009). At the core of the proposal is the key role of the main umami receptor. T1R1-T1R3 is very similar in architecture to the sweet receptor and it not only can recognize specifically some bitter L-amino acids but might eventually send a cross signal together with specialized bitter taste cells, according to the combinatorial model of taste coding (Roper and Chaudhari, 2017). This might explain why some L amino acids and aspartame diastereomers taste bitter.

When taken at face value, this hypothesis was interpreted to imply that all L-amino acids are bitter and, moreover, that they cannot be recognized by bitter receptors alongside the umami receptor (Meyerhof et al., 2015). This is not true, the original hypothesis by Temussi (2009) was meant simply as a way to draw attention on the possible role of the umami receptor in the bitter taste of some chiral isomers and on the interaction (cross talk) among different tastes. The ability of the sweet receptor to recognize D-Trp was recognized early on (Li et al., 2002). The detailed analysis of Meyerhof et al. (2015) confirms the findings of Bassoli et al. (2014) on the stereoselectivity of the sweet receptor, which can recognize essentially only D-amino acids, whereas only two aromatic L-amino acids, i.e., L-Trp and L-Phe, are recognized by bitter receptors. In mice most L-amino acids are recognized by the umami T1R1-T1R3 receptor (Li et al., 2002) whereas in humans, according to the prevailing view, only L-Glu and L-Asp are recognized and elicit an umami taste. This view is in conflict with the fact that T1R dimers have apparently evolved from common L-amino acid sensors (Nelson et al., 2002; Nuemket et al., 2017). More recently, the detailed study performed by Ninomyia and coworkers (Kawai et al., 2012) found that, at variance with the quoted report on human T1R1/T1R3 (Li et al., 2002), a wider variety of amino acids can elicit the umami taste.

These observations are not conclusive on the issue of a possible cross talk between umami and bitter tastes, which would suit the puzzling behavior of some L-/D- pairs, but open a possible new scenario for the interpretation of the diffuse occurrence of aftertaste in sweeteners, based on the key role of the T1R1-T1R3 receptor. Here we hypothesize that even when chirality is not involved, some sweet compounds may be easily recognized also by the umami receptor. This circumstance may have unwanted consequences on the quality of some sweeteners. Not only would some sweeteners have a bitter aftertaste but also some other taste quality. A sweetener tasting like beef broth may appeal to some cultures but to most human beings it will be perceived as an unpleasant or just *strange* taste quality.

**Figure 1:**
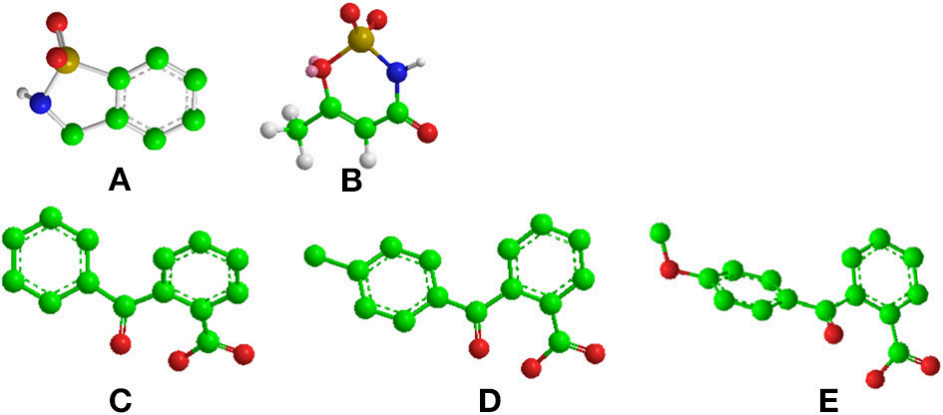
Chemical structure of **(A)** saccharin, **(B)** acesulfame, **(C)** 2-Benzoylbenzoic acid, **(D)** 2-(4-methylbenzoyl)benzoic acid and **(E)** 2-(4-methoxybenzoyl)benzoic acid, also known as S23_46 (Verkade, 1968). Carbon, oxygen, nitrogen and sulfur atoms are represented by filled green, red, blue and yellow circles, respectively. All models were built with Chem3D (trial version).

We checked the relative ability of sweet and umami receptors to bind *the same* (*achiral*) molecules, focusing on two common sweeteners (saccharin, acesulfame) and some arylcarbonylbenzoic acids with a marked aftertaste (Verkade, 1968). Molecular models of these sweeteners are reported in Figure 1. Docking calculations show conclusively that all of these sweeteners can be recognized also by the umami receptor with binding affinities similar to those found for the sweet receptor.

## Materials and Methods

### Homology Modeling of Taste Receptors

ClustalX (Thompson et al., 1997) was employed to get sequence alignments. Human T1R2-T1R3 and T1R1-T1R3 homology models were built with SWISS MODEL (Peitsch, 1995) in the hetero-oligomeric mode (Bertoni et al., 2017) using the crystal structure of the medaka fish receptor (PDB entry 5X2M) as template (Nuemket et al., 2017). NCBI accession numbers for the human sequences are: NP_619642.2 for hT1R1, AAM12239.1 for hT1R2 and NP_689414.2 for hT1R3. Subsequently, we checked independently the stereochemical quality of the models using Ramachandran plot (RAMPAGE), PROCHECK (Overall quality factor) and QMEAN (Lovell et al., 2003; Benkert et al., 2008). These models were visualized and rendered using the Visual Molecular Dynamics 1.9 (VMD) software Humphrey et al., 1996. The Ramachandran plots are shown in Figure S1.

### Ligand Preparation

The 3D structure of saccharin, acesulfame and 2-benzoylbenzoic acid used were obtained from the PubChem database, using the PubChem CIDs 5143, 36573, and 6813, respectively, whereas 2-(4-methylbenzoyl)benzoic acid and 2-(4-methoxylbenzoyl)benzoic acid were generated from the structure of 2-benzoylbenzoic acid using GaussView version 4.1. The generated structures were saved in mol2 file format.

### Molecular Docking of Sweeteners and Taste Receptors

Binding sites, as well as the associated free energies (ΔG_binding_), of the different sweeteners into sweet and umami taste receptors were predicted using Autodock Vina (Trott and Olson, 2009; Serio et al., 2014). Protein and ligand preparation was performed using Autodock Tools version 1.5.6 (Morris et al., 2009). Gasteiger partial charges were assigned to the atoms of ligands. The AutoTors option was used to define rotatable bonds in the sweeteners. The visual inspection of the results was performed using the MGL Tools package. Molecular docking was performed inside a volume of 70 × 70 × 90 grid points that comprises the whole taste receptors. The residues responsible for hydrogen bonding and hydrophobic interactions with sweeteners were identified and visualized using LigPlot+ (Wallace et al., 1995).

## Results

### Receptor Models

Our goal is to compare the ability of common sweeteners to interact with both the sweet and the umami receptor by calculating their docking energies with the corresponding homology models of the two receptors. To this end we need the structures of the two receptors. Unfortunately, neither has yet been determined experimentally. In the literature there are several homology models of the sweet receptor, built from the sequences of the receptors and several structures of similar class C GPCR receptors. Some homology models of the umami receptor have also been computed on a similar basis. However, we decided not to use any of the previous models but to take advantage of the recently published structure of the medaka fish “T1R2-T1R3” receptor (Nuemket et al., 2017). Formally, this is the first structure of a sweet taste receptor experimentally determined, but it is more reasonable to regard the taste receptor of fish medaka as an ancestor of both sweet and umami receptors of higher animals. The designation T1R2-T1R3, corresponding to the sweet receptor in mammals, apparently stems from genetic considerations (Nuemket et al., 2017). However, sequence comparisons using Clustal X (Thompson et al., 1997) show that the medaka fish taste receptor is more similar to the human umami receptor and indeed it was found that it recognizes L-amino acids, the hallmark of the T1R1-T1R3 receptor (the main human umami receptor), and not D- amino acids, the hallmark of the sweet receptor (Nuemket et al., 2017).

It is safer to consider it as the ancestor of both sweet and umami receptors in mammals.

We used this structure to build homology models of *human* umami and sweet receptors.

### Ligands

As hinted in the introduction, we wish to explore the possible role of the umami receptor in determining the quality of simple synthetic sweeteners. The choice of the sweeteners to test for a comparison between the sweet and umami receptors was kept to a small number of paradigmatic examples. We chose two of the best known (and used) synthetic sweeteners, i.e., saccharin and acesulfame. In addition we selected a few compounds from old literature (Verkade, 1968): 2_Benzoylbenzoic acid, 2-(4-methylbenzoyl)benzoic acid and 2-(4-methoxybenzoyl)benzoic acid. The latter compounds were well known for their sweetness accompanied by a marked aftertaste. All chosen ligands are essentially rigid from a conformational point of view.

### Docking

Among the many available docking programs, we chose “autodock VINA,” mainly because it is particularly suited for docking problems in which one of the partners is a protein whereas the ligand is a small molecule. We performed calculations using both protomers of each model, that is T1R1 of the umami, T1R2 of the sweet receptor and T1R3 protomers of both receptors. Considering that specificity is conferred to either receptor only by one protomer, T1R1 and T1R2 for umami and sweet respectively, from now on we show only the results for these two protomers.

The results for saccharin, acesulfame, 2_Benzoylbenzoic acid, 2-(4-methylbenzoyl)benzoic acid and 2-(4-methoxybenzoyl)benzoic acid, are summarized in Table 1. Corresponding data for the T1R3 protomers are reported in Table S1 (Supplementary Material). 2(4-methylbenzoyl)benzoic acid and 2-(4-methoxybenzoyl)benzoic acid have a ΔG_binding_ lower for both receptors than the rest of the compounds. In Table 1 are reported also the main amino acid residues of the protomer interacting with the ligands.

**Table 1:** Interaction energies and potential binding sites of protomers T1R2 and T1R1 for sweet and umami taste receptors.

**Ligand**	**hT1R2**		**hT1R1**	
	ΔG _*binding*_/kcal mole^−1^	Lining residues	ΔG _*binding*_/kcal mole^−1^	Lining residues
Saccharin	−5.1	N143, E145, D213, T242, N246, P277, L279, T280	−5.4	P45, S48, N69, H71, G72, D147, S276, R277
Acesulfame	−4.4	N143, E145, D213, T242, N246, P277, L279, T280	−5	P45, S48, N69, H71, G72, D147, S276
2_Benzoylbenzoic acid	−6.2	I104, N143, E145, D213, N246, P277, L279, T280	−6.2	P45, H47, S48, G49, N69, H71, G72, C106, D147, R151, S276, Q278
2–(4-methylbenzoyl) benzoic acid	−6.4	C102, Y103, I104, E145, T242, D213, N246, P277, L279, T280	−6.7	P45, H47, S48, N69, H71, L75, D147, A170, S276, R277, F381
2-(4-methoxybenzoyl) benzoic acid	−6.3	Y103, I104, E145, S211, T242, D213, N246, P277, L279, T280	−6.4	P45, S48, G49, N69, H71, G72, C106, D147, R151, S276, Q278

Before discussing in detail the results of the docking calculations it is in order to evaluate whether the values of the interaction energies are reliable. It is important in all calculations of this kind to keep in mind that, since the results depend on many factors that are beyond our control, such as the force field employed, it is important to anchor the results to experimental data if at all possible. In the cases at hand we do not have any data directly relating the figures of Table 1 to an experimental structure of complexes between the ligands and the receptor. However, it is possible to compare the values of free energy of interaction with the receptor to similar data backed by experimental results. For instance, it has recently been shown that it is possible to reproduce with great accuracy the position of an opioid inhibitor inside its receptor using Autodock Vina, the same program employed in the present work. The interaction energy calculated for naltrindole inside the delta opioid receptor was −13.6 kcal/mole for the lowest energy pose, identical to the crystallographic structure (Sanfelice and Temussi, 2014). Given that, as a rule of thumb, optimized interactions depend on the number of atoms and/or the molecular volume, it is fair to expect that saccharin (C7H5NO3S) may have an interaction energy approximately 0.4 times lower than that of naltrindole (C26H26N2O3), i.e., of *ca*. −5 kcal/mole.

The data summarized in Table 1 can also be interpreted in terms of consistency with previous results for sweet ligands. The only sweetener, among the ones chosen in the present paper, for which there have been calculations inside orthosteric active sites of different homology models of the sweet receptor is saccharin.

As reported by Morini and Temussi (2005) the interaction energy with the T1R2 active site, calculated by the program PrGen (Vedani et al., 1993) is −9.6 kcal/mole. This value is larger in absolute value than that reported in Table 1. However, the value reported by Morini and Temussi (2005), apart from being calculated using a quite different force field, was biased by the inclusion of saccharin in the training set of the calculation. In other words, the need of forcing a set of several ligands to agree with experimental data drove single values toward lower figures. In addition, the site used by Morini and Temussi (2005) was an open site whereas that of the present model is closed as in the experimental structure of the medaka fish. All things considered it is fair to regard the present value consistent with the literature data. Accordingly, all energy figures reported in Table 1 for the sweet receptor appear reliable.

Another important aspect is the consistency of the residues lining the T1R2 active site in the present docking with respect to those found in previous studies using different homology models and different methodologies. On the basis of multiple alignments of GPCR sequences, it was predicted by Morini and Temussi (2005) that the most likely residues of the active site on the hT1R2 protomer ought to be N143, S144, S165, I167, A187, Y215, P277, D278, and E302. The prominent residues interacting with saccharin that we find in our site modeled after the medaka fish structure are N143, E145, D213, T242, N246, P277, and T280. Between N143 and P277 there are three more residues (E145 D213 L279) very close in sequence to S144, Y215, and P277, respectively. It is fair to say that the two active site occupy a similar space in the receptor structure.

**Figure 2:**
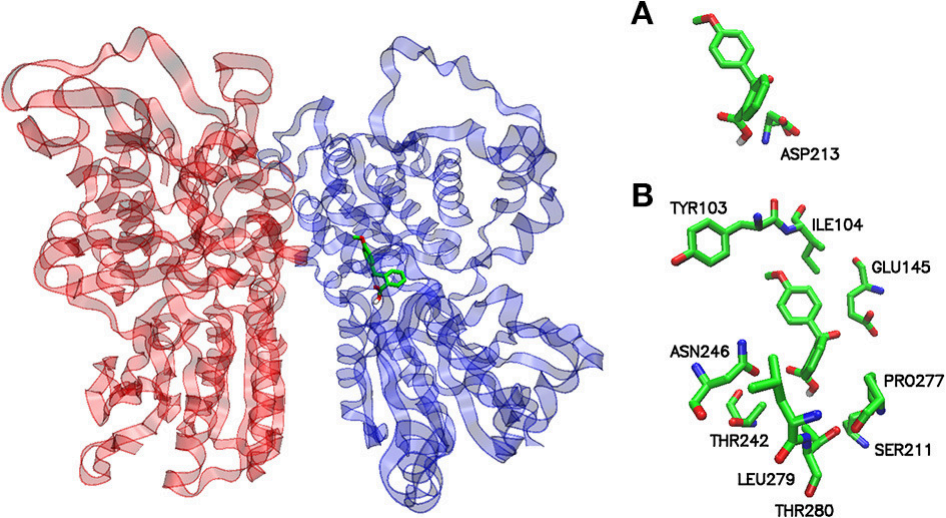
Potential binding site for the pose of lowest energy of docked 2-(4-methoxybenzoyl) benzoic acid within the sweet taste receptor. Ribbon representation of the T1R2-T1R3 homology model with a stick model of the sweet ligand inside the active site (left panel). Protomers T1R2 and T1R3 are colored blue and red, respectively. A detailed inspection of the binding site within hT1R2 shows the amino acids responsible for the interaction with 2-(4-methoxybenzoyl)benzoic acid (right panel). **(A)** hydrogen bond and **(B)** hydrophobic contacts, respectively. The models were generated by VMD.

**Figure 3:**
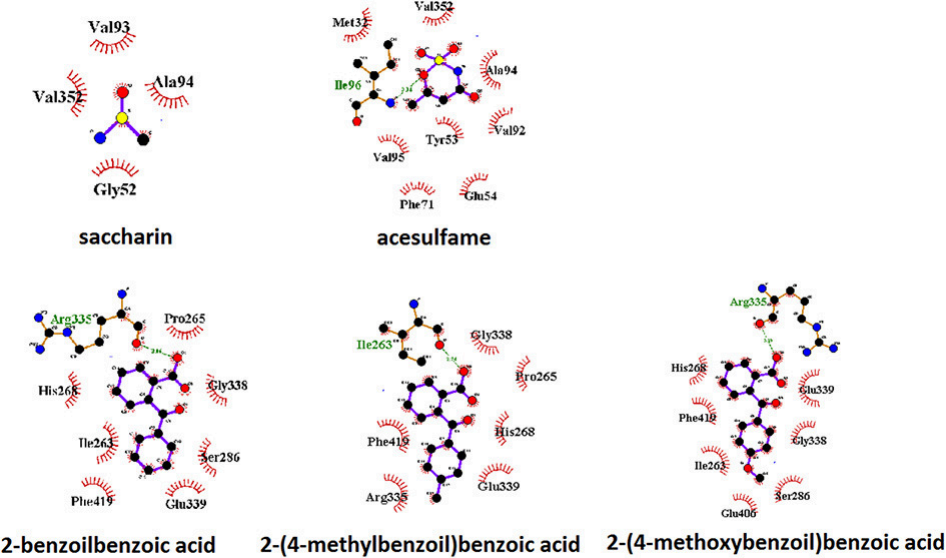
Pattern of binding of sweeteners with the sweet taste receptor. The amino acids responsible for the hydrogen bonds and hydrophobic interactions are represented by three-letter codes in green and black, respectively. Carbon, oxygen, and nitrogen atoms are represented by filled black, red, and blue circles, respectively.

A view of the complex between the receptor model and 2-(4-methoxybenzoyl)benzoic acid, the sweetener with the best affinity among those calculated in the present work, is shown in Figure 2. The role of residues lining the walls of the active site is interpreted in terms of potential hydrogen bonds and hydrophobic contacts. A bird's eye view of the interactions of all ligands studied here with the residues of the active site of the human sweet receptor can be seen in Figure 3. Not unexpectedly, the arrangement of residues around acesulfame is very similar to that around saccharin, a clear consequence of the similarity of chemical constitution between the two compounds. The number of residues interacting with the benzoic acids reflect the higher number of atoms in these ligands. The slightly different patterns observed for the three benzoic acids speak of the versatility of the sweet taste receptor; yet seven out of eight residues lining saccharin are found also for 2-benzoylbenzoic acid.

**Figure 4:**
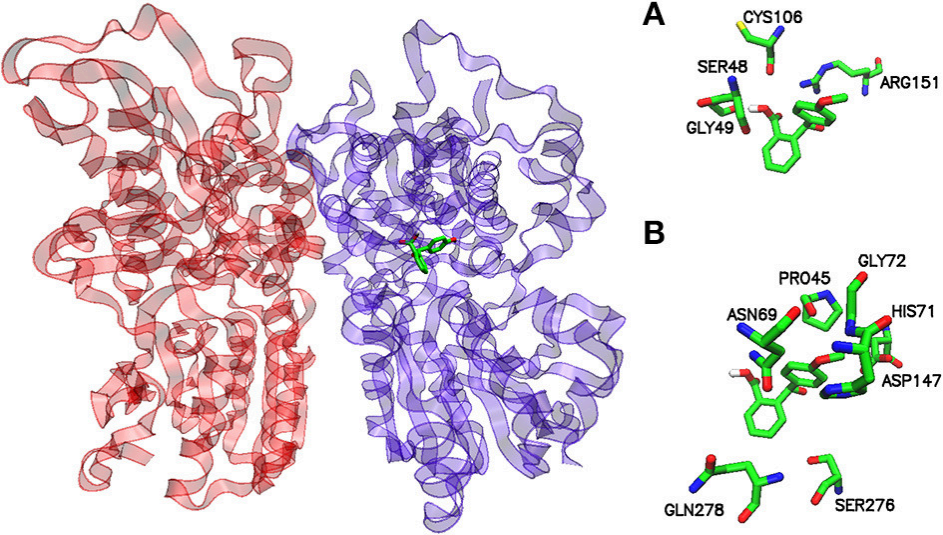
Potential binding site for the pose of lowest energy of docked 2-(4-methoxybenzoyl) benzoic acid within the umami taste receptor. Ribbon representation of the T1R1-T1R3 homology model with a stick model of the sweet ligand inside the active site (left). Protomers T1R1 and T1R3 are colored violet and red, respectively. A detailed inspection of the binding site within hT1R1 shows the amino acids responsible for the interaction with 2-(4-methoxybenzoyl)benzoic acid (right). **(A)** hydrogen bond and **(B)** hydrophobic contacts, respectively. The models were generated by VMD.

**Figure 5:**
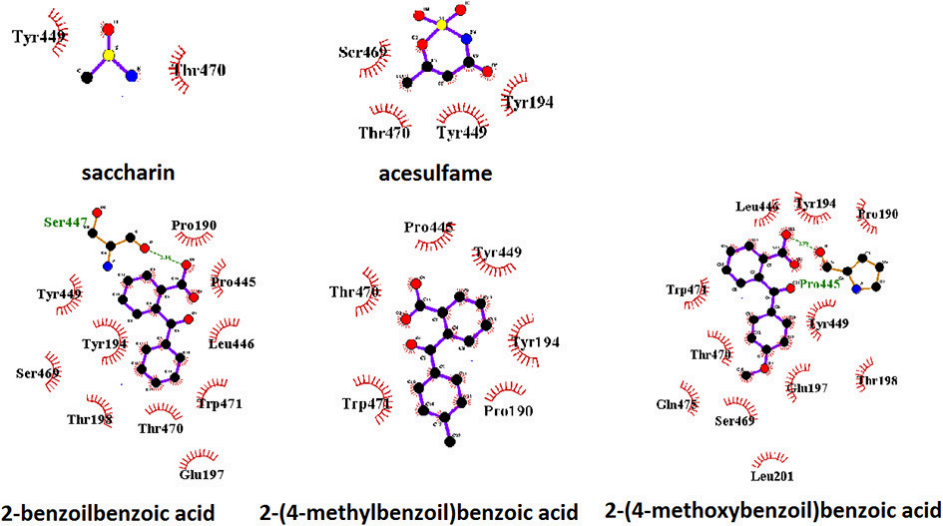
Pattern of binding of sweeteners with the umami taste receptor. The amino acids responsible for the hydrogen bonds and hydrophobic interactions are represented by three-letter codes in green and black, respectively. Carbon, oxygen, and nitrogen atoms are represented by filled black, red, and blue circles, respectively.

A parallel representation for the umami receptor is illustrated in Figures 4, 5.

A view of the complex between the umami receptor model and 2-(4-methoxybenzoyl)benzoic acid, the ligand with the best affinity among those calculated in the present work, is shown in Figure 4. The role of residues lining the walls of the active site is interpreted in terms of potential hydrogen bonds and hydrophobic contacts. A bird's eye view of the interactions of all ligands with the residues of the umami active site can be seen in Figure 5. Similarly to what found for the sweet receptor, the arrangement of residues around acesulfame is very close to that around saccharin, a clear consequence of the similarity of chemical constitution between the two compounds.

Also for the interactions of the umami receptor with ligands chosen in the present work it is possible to state that the residues lining the active site are very similar for all ligands.

Altogether it is fair to say that the ability of both receptors to interact with the chosen tastants is comparable. Should one take at face value the data of Table 1, it would appear that the ligands interact more strongly with the umami receptor. Such a view does not take into account the limitations of simulations with respect to experimental data. Our calculations hint at the similarity of the interactions with two receptors but the *in vivo* experience with the tastants tells us that the main response in humans is for sweet substances. This fact may well depend on kinetic factors rather than on equilibrium interaction energies. However, the data of Table 1 do tell us that the umami receptor can contribute to the final taste adding unpleasant aftertastes either directly as umami taste or indirectly as bitter taste via a cross talk mechanism because the affinity of all ligands for the umami receptor is comparable to those for the sweet receptor.

## Discussion

The idea that the human umami receptor can play a direct role in the recognition of bitter isomers of pairs of sweet/bitter isomers cannot be dismissed altogether, as long as the dogma of completely labeled taste cells can be taken as a possibility consistent with alternative visions, as the combinatorial model proposed by Roper and Chaudhari (2017). In addition, even if the contribution of the umami receptor via cross talk with bitter taste cells is not accepted, it is clear from the results presented here that the T1R1-T1R3 receptor plays a key role in modulating the quality of sweet tastants.

## Author Contributions

WA performed all calculations and contributed to writing; PT proposed the hypothesis and made most of writing.

### Conflict of Interest Statement

The authors declare that the research was conducted in the absence of any commercial or financial relationships that could be construed as a potential conflict of interest.
