# Supplementary material for: The Origin of Unpleasant Aftertastes in Synthetic Sweeteners: A Hypothesis
Source: Front Mol Biosci. 2019 Jan 18;5:119. doi: 10.3389/fmolb.2018.00119 (PMC6345712; doi:10.3389/fmolb.2018.00119)
Supplement: Supplementary file 1 [file Data_Sheet_1.PDF]

The origin of unpleasant aftertastes in synthetic sweeteners

Waldo Acevedo and Piero Andrea Temussi

SUPPLEMENTARY MATERIAL

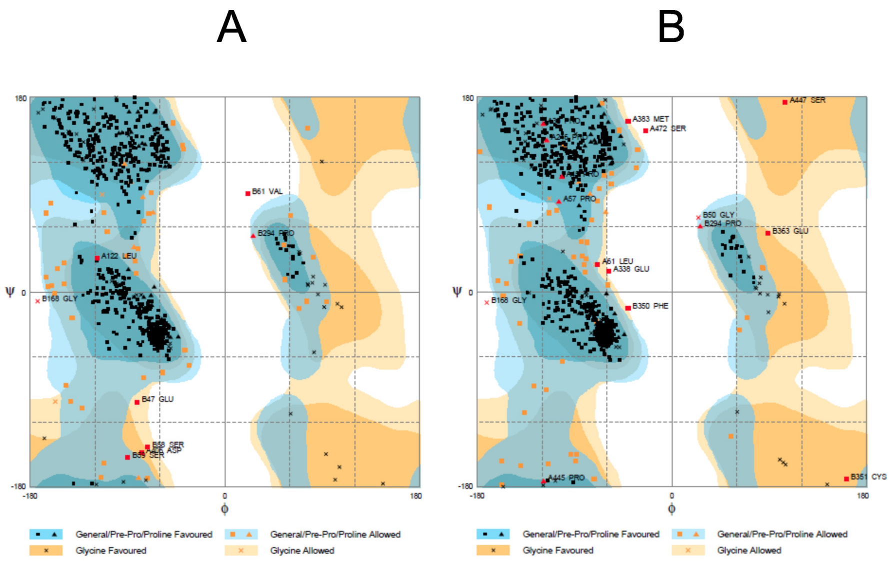

Figure S1. Ramachandran maps of the homology models for the sweet (A) and umami (B) receptors. Ramachandran plots indicate that 92.0% of the residues are in favoured regions, 6.2% in allowed regions and 1.9% in outlier regions for umami receptor; whereas 93.1% of the residues are in favoured regions, 5.9% in allowed regions, and 1.0% in outlier regions for sweet receptor. PROCHECK analyses of the umami and sweet models yielded overall average G factors of -0.37 and -0.32, respectively, which suggest that the conformations both, of main and side chain of the models, are reasonable. The value of QMEAN score for the umami and sweet receptor were -3.35 and -1.82, respectively, pointing that the resulting models were close enough to a set of experimental protein structures from the PDB database.

Table S1. Interaction Energies and Potential Binding Sites for T1R3 protomers of Sweet and Umami Taste Receptors.

ligand	HT1R3 (sweet)		HT1R3 (umami)	
	$\Delta G_{binding}$/ kcal mole^{-1}	lining residues	$\Delta G_{binding}$/ kcal mole^{-1}	lining residues
saccharin	-5.8	H145, S146, G168, Y218, S276, V277, A302	-5.8	H145, S146, G168, Y218, S276, V277, A302
acesulfame	-5	H145, S146, Y218, S276, V277, A302	-5	H145, S146, Y218, S276, V277, A302
2_Benzoylbenzoic acid	-6.7	P42, E45, W72, H145, G168, Y218, S276, V277, A302	-6.7	P42, E45, S66, W72, H145, S146, G168, Y218, S276, V277, A302
2-(4-methylbenzoyl) benzoic acid	-6.9	S66, W72, H145, S146, S147, G168, Y218, S276, V277, A302	-6.8	E45, S66, W72, H145, S146, S147, G168, Y218, S276, V277, A302
2-(4-methoxybenzoyl) benzoic acid	-6.8	P42, E45, S66, W72, H145, S146, S147, G168, Y218, S276, V277, A302	-6.7	P42, E45, S66, W72, H145, S146, S147, G168, Y218, S276, V277, A302
